# Subsampling Approaches for Compressed Sensing with Ultrasound Arrays in Non-Destructive Testing

**DOI:** 10.3390/s20236734

**Published:** 2020-11-25

**Authors:** Eduardo Pérez, Jan Kirchhof, Fabian Krieg, Florian Römer

**Affiliations:** 1 Fraunhofer IZFP, 66123 Saarbrücken, Germany; jan.lukas.kirchhof@izfp.fraunhofer.de (J.K.); fabian.krieg@izfp.fraunhofer.de (F.K.); florian.roemer@izfp.fraunhofer.de (F.R.); 2 Electronic Measurements and Signal Processing, TU Ilmenau, 98693 Ilmenau, Germany; 3 AutomaTiQ, htw saar, 66117 Saarbrücken, Germany

**Keywords:** Full Matrix Capture, compressed sensing, sparse array

## Abstract

Full Matrix Capture is a multi-channel data acquisition method which enables flexible, high resolution imaging using ultrasound arrays. However, the measurement time and data volume are increased considerably. Both of these costs can be circumvented via compressed sensing, which exploits prior knowledge of the underlying model and its sparsity to reduce the amount of data needed to produce a high resolution image. In order to design compression matrices that are physically realizable without sophisticated hardware constraints, structured subsampling patterns are designed and evaluated in this work. The design is based on the analysis of the Cramér–Rao Bound of a single scatterer in a homogeneous, isotropic medium. A numerical comparison of the point spread functions obtained with different compression matrices and the Fast Iterative Shrinkage/Thresholding Algorithm shows that the best performance is achieved when each transmit event can use a different subset of receiving elements and each receiving element uses a different section of the echo signal spectrum. Such a design has the advantage of outperforming other structured patterns to the extent that suboptimal selection matrices provide a good performance and can be efficiently computed with greedy approaches.

## 1. Introduction

Multi-channel ultrasound inspection techniques enable high resolution imaging by exploiting spatial information collected with an array. Commonly employed multi-channel measurement modalities include Full Matrix Capture (FMC) in non-destructive evaluation [1] and Plane Wave Imaging (PWI) in medical applications [2]. The resolution of such techniques is linked to the number of transmission cycles and is therefore inversely proportional the overall measurement speed. The focus of this work is the design of subsampling patterns for FMC that result in a reduction of both measurement times and data volumes. The study is presented from a compressed sensing standpoint [3]; as such, imaging is treated as a regularized inverse problem and the design of the subsampling patterns yields the corresponding compression matrix.

Sparse recovery and compressed sensing have been successfully applied to ultrasound imaging, where improved image quality and large compression rates have made them attractive approaches. In [4], reconstructions from a single plane wave are shown to be comparable to those from synthetic aperture focusing when the measurement scenario is simple and sparse recovery is used. The standard Total Focusing Method (TFM) described in [1] is treated as a sparse recovery problem in [5], where it results in an enhanced resolution. The same authors go on to reformulate the sparse recovery problem directly in the domain of the reconstructed image in [6], speeding up the computation time. Compressed beamforming is presented in [7], where images are reconstructed from signals sampled below the Nyquist rate based on their finite rate of innovation. In [8], a wave atom dictionary is employed in order to recover the original measurement data prior to beamforming. Sparse recovery is carried out on Ultrasound Computed Tomography (UCT) data using dictionaries learned from previously processed data sets in [9].

Different approaches to measurement speed enhancement via compressed sensing can be found in the ultrasound imaging literature. UCT is sped up in [10] by allowing multiple transducers to transmit with random amplitudes. Such an approach yields high imaging quality by virtue of a Signal to Noise Ratio (SNR) that stems from the simultaneous transmission events [1]. A contrasting approach that does not necessitate simultaneous transmissions and arbitrary amplitude manipulations is to simply leave out measurement samples. Reconstructions from such subsampled 3D array data are studied in [11,12], where it is shown that complete data scans can be omitted with only a small increase in the error. Indeed, it was shown in [13] that high quality reconstructions can be obtained even when the FMC data has been heavily subsampled both spatially (i.e., by omitting the data from several sensors) and in the frequency domain.

Spatial subsampling is closely related to sparse array design and optimal placement of sensors, which are topics of widespread interest and applicability. Sparse arrays have been studied extensively via the design of cost functions that incorporate the main and side lobes produced by the array, followed by optimization with methods like simulated annealing [14] or genetic algorithms [15]. The optimal position of a predefined number of sensors can be sought after in a similar fashion. To this end, in the field of acoustics recent works have focused on the greedy optimization of cost functions such as the mutual information between sensor locations that have already been assigned and those that are still open [16], or the average size of the main lobe in a Region of Interest (ROI) over which the probability of finding a source is non-uniform [17]. When paired with convolutional beamforming, the elements in a pair of co-prime arrays may also be placed such that the resulting co-array has a larger aperture and effectively contains all of its elements [18].

The problems of optimal sensor placement and spatial and frequency domain subsampling can be addressed together with compressed sensing by incorporating them into the design of the compression matrix. In this vein, co-prime arrays have been designed from closed-form expressions for Direction of Arrival (DOA) estimation paired with sparse recovery using a steering vector dictionary [19]. In [20], the compression matrix for MRI imaging is designed by estimating the best probability density function from which coefficients should be drawn. In this work, sparse array design is dealt with by studying the impact the resulting compression matrix has on the CRB of a point-like scatterer whose coordinates are to be estimated.

The CRB is a lower bound on the variance of an estimated parameter that an unbiased estimator can achieve [21]. It is estimator agnostic and depends only on the data model and its parameters. In [22], the CRB and Monte Carlo simulations were employed to study the resolution limit of ultrasound localization microscopy, showing a direct relationship to the aperture size and therefore the spacing between the active sensors. This behavior was quantified in [23], where approximations and simplifications allowed the CRB of FMC data containing echoes from a single scatterer to be written in a closed form that clearly portrayed the impact of the sensor positioning on the variance of the estimated scatterer location. This insight was then used to design a channel selection technique with the CRB as a cost function. Interestingly, a similar approach is followed in [24], in which the CRB of a set of points is used in order to derive a closed form expression for the optimal position and orientation of cameras for 3D vision. In addition, combining matrices for DOA estimation have also been designed by considering the CRB and probability of false detection in [25].

Motivated by the success of compressed sensing in ultrasound imaging and the ubiquity of sparse array design and optimal sensor positioning problems, the present study expands upon the results in [13,23]. By enforcing specific conditions on the transmitting and receiving elements in a Uniform Linear Array (ULA), structured compression matrices are designed based on the CRB of a single scatterer whose location is estimated from a subsampled FMC measurement. These compression matrices represent sparse arrays with subsampled spectra that allow the fast collection of subsampled FMC data from which high resolution reconstructions may be obtained. In [13], strict conditions were enforced on the compression matrix: each transmitter must use the same subset of receivers and every channel uses the same section of the signal bandwidth. In this paper, a more flexible approach is studied for spatial and frequency subsampling. In particular, each active transmitter is only required to have the same number of associated receivers, while each transmitter-receiver pair must have the same number of frequency samples; however, the actual elements and frequency samples can be chosen freely. Since the presented novel approaches generalize the previous work [13], they entail a higher computational complexity. Therefore, this work also adapts the compression matrix design approach from [23] into a two stage greedy algorithm. The resulting imaging quality is then compared to that in [13] by applying the new compression matrices to the same measurement data. Additionally, the previous and present approaches are contrasted in new synthetic scenarios.

## 2. Full Matrix Capture

**Figure 1:**
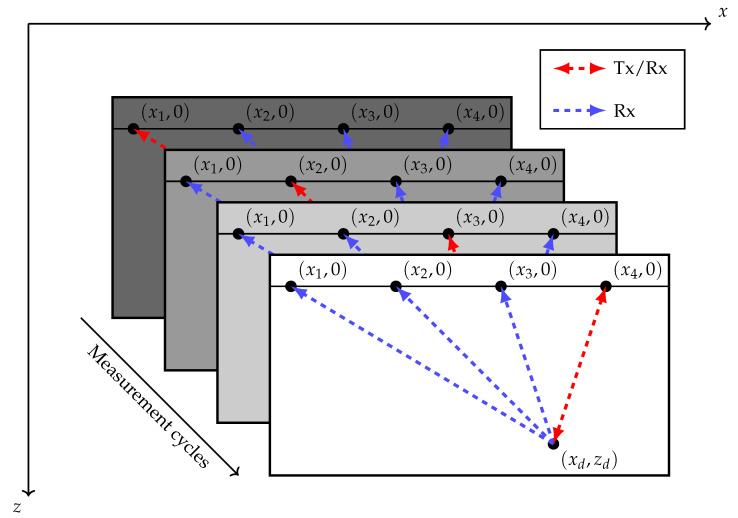
Illustration of FMC measurement. A ULA whose centroid lies at (x,z)=(0,0) emits pulses into the medium sequentially. Reflections from a flaw located at $x_{d},z_{d}$ are observed in each measurement cycle.

FMC is a multi-channel measurement technique in which the signals from all possible combinations of transmitting (Tx) and receiving (Rx) elements are collected [1]. A sketch of this procedure is presented in Figure 1, in which a scenario with a simple measurement geometry is shown. The main idea behind FMC is to sequentially excite the specimen with each element in an array. After each transmission, the Rx elements record the echoes produced at impedance discontinuities within the medium. Each Tx event is dubbed a measurement cycle due to the possibility of parallel Rx recording. In the case of a ULA placed on a flat surface, the depth coordinate of the sensors can be taken as z=0 mm without loss of generality.

The measurement cycles axis in Figure 1 is analogous to time, making it evident that the duration of the measurement procedure scales as *M* for a ULA with *M* elements. A straightforward way of speeding up the procedure is to reduce the number of Tx events (i.e., measurement cycles). The classical approach for data acquisition and imaging would impose a trade-off between the number of cycles (speed) and the aperture size (resolution). By using subsampling, this trade-off can be circumvented. As the goal is to perform a parametric reconstruction of the underlying image using compressed sensing, it is desirable to model the FMC data in such a way that the reduction of Tx and Rx events can be represented through compression matrices. Before this, however, a compatible model is necessary.

### 2.1. Data Model

**Figure 2:**
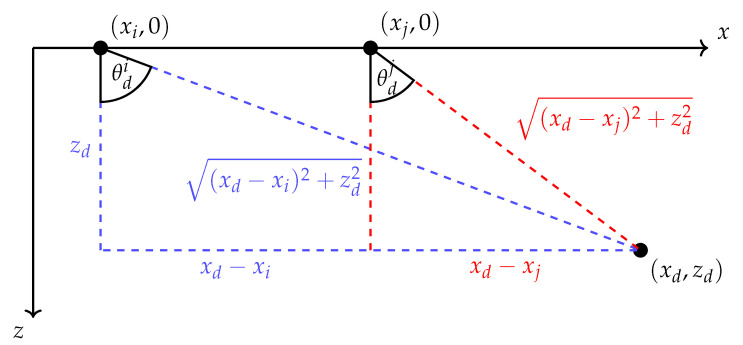
Illustration of the geometric relationships among a pair of sensors and a scatterer. These relationships completely determine the time of flight and angles of departure and arrival in a homogeneous, isotropic medium. The figure has been adapted from [13,23] to reflect changes in notation.

The same data model as in [13,23] is used throughout this work. This model is based on the first order Born approximation, which considers the scattered field resulting only from the incident field. In a homogeneous and isotropic 2D setting with point-like scatterers and no dispersion, a bistatic noiseless measurement can be modeled as

(1) $$b_{i,j}^{\left(a\right)}\left(t\right)=\left(\sum_{d=1}^{D}a_{d}\;\cdot \;\exp\left(j\phi_{d}\right)\cdot g_{i,j}\left(\theta_{d}^{i},\theta_{d}^{j}\right)\;\cdot \;\delta \left(t-\tau_{i,j}\left(x_{d},z_{d}\right)\right)\right)*h^{\left(a\right)}\left(t\right),$$

where the subindices i,j denote the Rx and Tx elements, respectively. The pulse shape is denoted as $h^{\left(a\right)}\left(t\right)$, with the superscript (a) indicating the analytic representation. It is assumed that the pulse shape is generated by the Tx element and remains unchanged at the Rx side. Additionally, ∗ represents the convolution of two functions. The number of scatterers in the medium is denoted as *D*, which in the interest of compressed sensing is assumed to be small. Each scatterer *d* has an associated complex weight $a_{d}\;\cdot \;\exp\left(j\phi_{d}\right)$ that determines its scattering amplitude and phase. The term $g_{i,j}\left(\theta_{d}^{i},\theta_{d}^{j}\right)$ accounts for the directivities of the Tx and Rx elements which respectively depend on the Direction of Departure (DOD) $\theta_{d}^{j}$ and Direction of Arrival (DOA) $\theta_{d}^{i}$. The term δ(·) represents the delta function, and $\tau_{i,j}\left(x_{d},z_{d}\right)$ is the time delay for scatterer *s* located at $\left(x_{d},z_{d}\right)$ for the channel corresponding to Tx element *j* and Rx element *i*. These geometric relationships are shown in Figure 2. For simplicity, a Gaussian directivity is assumed both in Tx and Rx following

(2) $$\begin{array}{c} g_{i,j}\left(\theta_{d}^{i},\theta_{d}^{j}\right)=\exp\left(\frac{-\tan^{2}\left(\theta_{d}^{j}\right)-\tan^{2}\left(\theta_{d}^{i}\right)}{\tan^{2}\left(\theta_{0}\right)}\right), \end{array}$$

with $\theta_{0}$ as the transducer’s opening angle. However, the model and the presented subsampling strategies admit any directivity function that can be differentiated with respect to the scatterer location $x_{d},z_{d}$, amplitude $a_{d}$, and phase $\phi_{d}$.

If a ULA with *M* elements is used, a total of $M^{2}$ bistatic measurements obeying (1) are comprised in the FMC data. Each single-channel scan is sampled at a rate $f_{s}$ yielding $N_{T}$ samples, so that measurement vectors $b_{i,j}^{\left(a\right)}\in C^{N_{T}}$ are obtained. Finally, the measurements can be concatenated as

(3) $$b={\left[{\left(b_{1,1}^{\left(a\right)}\right)}^{T},\;{\left(b_{2,1}^{\left(a\right)}\right)}^{T},\;\cdots ,\;{\left(b_{M,M}^{\left(a\right)}\right)}^{T}\right]}^{T}\in C^{N_{T}\;\cdot \;M^{2}}.$$

### 2.2. Discrete Forward Model

A forward model for b is formulated next. To begin, consider the discretization of (1) defined in (3). The first operation to be discretized is the convolution with the pulse shape $h^{\left(a\right)}\left(t\right)$. This is done by defining a Toeplitz matrix $H\in C^{N_{T}\times N_{T}}$ that contains shifted copies of the time sampled pulse shape along its columns. Since the model is a superposition of hyperboloids, a dictionary containing the possible time delays is constructed. A ROI is discretized with some horizontal and vertical resolution $\Delta_{x}$ and $\Delta_{z}$, noting that this discretization inevitably introduces an estimation error directly related to the chosen resolution [26]. The region can then be modeled as a vector $x\in C^{N_{z}\;\cdot \;N_{x}}$, where $N_{x}$ and $N_{z}$ denote the number of samples along the horizontal and vertical directions. The elements of $x_{n}$ of x represent the complex weights of potential flaws in a specimen (i.e., the reflectivity of the measured specimen) while their index *n* indicates the location within the ROI. A forward model is built in such a way that the non-zero elements in x are mapped into hyperboloids in b. For simplicity, the forward model is first defined for a single channel as

(4) $$b_{i,j}^{\left(a\right)}={\mathrm{HM}}_{i,j}x.$$

The matrix $M_{i,j}\in R^{N_{T}\times N_{z}\;\cdot \;N_{x}}$ represents the time delays and directivities corresponding to the potential scatterer locations in x, which are then convolved with the pulse shape through a product with H. Replacing the original dependence on $x_{d},z_{d}$ by a dependence on the elements $x_{n}$ of x, the components of the time delay matrix can be computed following

(5) $${\left(M_{i,j}\right)}_{m,n}=g_{i,j}\left(\theta_{x_{n}}^{i},\theta_{x_{n}}^{j}\right)\;\cdot \;\delta \left[m-\left\lfloor f_{s}\;\cdot \;\tau_{i,j}\left(x_{n}\right)\right\rfloor \right],$$

where δ[·] is the Kronecker delta and ⌊·⌋ is the floor function, and the parentheses around $M_{i,j}$ are used to distinguish the channel subindices i,j from the indices m,n of the matrix elements. Using (5), the complete time delay dictionary for b is given by

(6) $$M={\left[M_{1,1}^{T},\;M_{2,1}^{T},\;\cdots ,\;M_{M,M}^{T}\right]}^{T}\in R^{N_{T}\;\cdot \;M^{2}\times N_{z}\;\cdot \;N_{x}}$$

following the same stacking procedure as in (3). Using $I_{N}\in R^{N\times N}$ as the identity matrix whose size is specified by its subscript, the pulse shape matrix is multiplied individually to each channel via

(7) $$b=(I_{M^{2}}⊗H)\mathrm{Mx}=\mathrm{Ax},$$

thus modeling a complete FMC data set.

## 3. Subsampled Data

The effort in sparse array design and optimal sensor position is largely dedicated to choosing a performance metric that promotes the desired imaging behavior. These metrics include, but are not limited to, beam patterns [14,15,17], number of virtual sensors [18,19], mutual coherence [8], information-theoretic quantities [16], and data-driven quantities [20]. Similarly to [22], the approach in this paper focuses on minimizing the variance of the estimate of the location of point-like scatterers. In contrast to [22], the variance is calculated analitically, as opposed to computing it from Monte Carlo simulations. More specifically, a lower bound on the variance, i.e., the CRB [21], of a single point-like scatterer is considered. This requires the model to incorporate subsampling directly, and so an appropriate extension is presented next.

In the spirit of compressed sensing, the action of only collecting data from a small set of channels is represented as a product with a compression matrix. Such a matrix can consider not only spatial, but also frequency domain subsampling of the data. It should be noted that only Tx subsampling yields a gain in measurement time; however, Rx and frequency subsampling can further compress the data and make it feasible to inspect larger specimens and store the data therefrom. Reconstructions can be carried out with only a small number of Fourier coefficients per channel, as shown in [7,27]. This can be done in several ways; however, focus is restricted to two structures which result in a smaller number of measurement cycles without requiring a specialized hardware beyond programmable switches and a means to compute fast Fourier transforms per channel.

The first of these approaches employs a single set of transmitters, receivers, and Fourier coefficients as defined in [13,23]. The transmitters are chosen with a selection matrix $S_{T}\in R^{M_{T}\times M}$ that chooses $M_{T}<M$ elements in the ULA. Likewise, the matrices $S_{R}\in R^{M_{R}\times M}$ and $F\in C^{N_{F}\times N_{T}}$ select $M_{R}<M$ Rx elements and $N_{F}<N_{T}$ Fourier coefficients respectively. Note that the total number of Fourier coefficients is $N_{T}$ if a usual Discrete Fourier Transform (DFT) matrix used, but since the signals are analytic, half of the coefficients are zero and only $\frac{N_{T}}{2}$ coefficients remain. Of these, $N_{F}<\frac{N_{T}}{2}$ coefficients are chosen by keeping the corresponding subset of rows from the DFT matrix in F. In this model, every channel collects the same set of Fourier coefficients. Additionally, the same set of Rx elements is active during each measurement cycle. An FMC data set subsampled in this fashion is given by [13,23]

(8) $$\begin{array}{c} y_{K}=\left(S_{T}⊗S_{R}⊗F\right)b+n_{K}\in C^{N_{F}\;\cdot \;M_{R}\;\cdot \;M_{T}}, \end{array}$$

with ⊗ denoting the Kronecker product and $S_{K}=\left(S_{T}⊗S_{R}⊗F\right)\in C^{N_{F}\;\cdot \;M_{R}\;\cdot \;M_{T}\times N_{T}\;\cdot \;M^{2}}$ as the compression matrix. In (8), the term $n_{K}$ has been added to account for noise added to b upon measurement, meaning it has also been compressed. Throughout this work, it is assumed that the measurement noise is zero-mean, circularly symmetric, white (both in space and time) Gaussian noise. The subsampling operation $\left(S_{T}⊗S_{R}⊗F\right)$ has orthogonal rows and therefore preserves the noise statistics, although the variance is modified depending on the normalization choice for F. For simplicity, the frequency subsampling matrices are left unnormalized, which results in an energy scaling factor of $\frac{N_{T}}{2}$ after considering that the signals are analytic.

The second approach allows more flexibility. Now, each Tx element has its own set of $M_{R}$ receivers. Further, each channel has its own, possibly unique, set of $N_{F}$ Fourier coefficients. The corresponding subsampled data set can be modeled as

(9) $$\begin{array}{c} y_{KR}=\left(\left(\left(S_{T}⊗{𝟙}_{M}^{T}\right)⋄\left[S_{R1},\;S_{R2},\;\cdots ,\;S_{RM}\right]\right)⊗{𝟙}_{N_{T}}^{T}\right)⋄\left[F_{1},\;F_{2},\;\cdots ,\;F_{M^{2}}\right]b+n_{KR}\in C^{N_{F}\;\cdot \;M_{R}\;\cdot \;M_{T}}, \end{array}$$

where ⋄ is the Khatri–Rao or column-wise Kronecker product and ${𝟙}_{N}\in R^{N}$ is a vector of ones whose dimension is indicated in the subscript. The matrix $\left[S_{R1},\;S_{R2},\;\cdots ,\;S_{RM}\right]\in R^{M_{R}\times M^{2}}$ is a concatenation of *M* Rx selection matrices. In a similar fashion, $\left[F_{1},\;F_{2},\;\cdots ,\;F_{M^{2}}\right]\in C^{N_{F}\times N_{T}\;\cdot \;M^{2}}$ is a concatenation of $M^{2}$ Fourier selection matrices. Although this notation requires the definition of several $S_{Ri}$ and $F_{i}$ matrices with only zeros, it highlights the underlying structure of the compression matrix $S_{KR}\;=\;\left(\left(\left(S_{T}⊗{𝟙}_{M}^{T}\right)⋄\left[S_{R1},\;S_{R2},\;\cdots ,\;S_{RM}\right]\right)⊗{𝟙}_{N_{T}}^{T}\right)⋄\left[F_{1},\;F_{2},\;\cdots ,\;F_{M^{2}}\right]\in C^{N_{F}\;\cdot \;M_{R}\;\cdot \;M_{T}\times N_{T}\;\cdot \;M^{2}}$. Once again, the term $n_{KR}$ is zero-mean, circularly symmetric, white Gaussian noise.

The subsampling approach in (9) is more flexible than that of (8). It is important to highlight that the operations involving the Fourier subsampling matrices $F_{i}$ can be carried out in the same way in both settings, and they are equivalent if $F_{i}=F$. In fact, the subsampling model (8) from [13,23] is a special case of (9) in which $F_{i}=F$ and $S_{Ri}=S_{R}$. The new model (9) adopted in this work generalizes (8) by decoupling the subsampling choice for the transmission and reception elements, as well as the selection of frequency coefficients. This opens up the possibility of combining different spatial and frequency subsampling approaches, and so a different naming scheme is adopted. The spatial subsampling approach in (8) is referred to as constant Rx, since the same set of Rx elements is used in every measurement cycle. In contrast, the approach in (9) is called varying Rx from here on, as it allows the possibility of using different receivers (albeit the same amount thereof) in each cycle. Fourier subsampling requires further discussion and is therefore addressed in Section 6.3.

## 4. Compressed Sensing

### 4.1. Problem Description

The theory of compressed sensing brings to light the possibility of reconstructing vectors from a small number of measurement samples if an appropriate sparsifying dictionary is known [3]. Recall (1), in which FMC data is modeled as a sum of scaled, phase shifted, and time delayed pulses. The time delays $\tau_{i,j}\left(x_{d},z_{d}\right)$ trace a slowly-varying hyperbolic shape across space (i.e., across the sensors) and the energy of the signal is concentrated near the center frequency of the pulse shape $h^{\left(a\right)}\left(t\right)$. This hints at a sparse representation based on these two properties that can be readily used to formulate the imaging task as a compressed sensing problem. Similarly to the approach taken in [5,6], imaging is treated as an inverse problem with sparse regularization. The addition of compression matrices turns it into a standard compressed sensing problem, as shown next. The subsampling scheme (9) is used in the following formulation, as it encompasses (8) as a special case.

Frequency subsampling is considered first, which is addressed by the term

(10) $$S_{f}=\left[F_{1},\;F_{2},\;\cdots ,\;F_{M^{2}}\right]\in C^{N_{F}\times N_{T}\;\cdot \;M^{2}}.$$

Spatial subsampling is represented via the matrix

(11) $$S_{s}=\left(\left(\left(S_{T}⊗{𝟙}_{M}^{T}\right)⋄\left[S_{R1},\;S_{R2},\;\cdots ,\;S_{RM}\right]\right)⊗{𝟙}_{N_{T}}^{T}\right)\in R^{M_{R}\;\cdot \;M_{T}\times N_{T}\;\cdot \;M^{2}}.$$

The overall compression matrix is then given by

(12) $$\Phi =S_{s}⋄S_{f}\in C^{N_{F}\;\cdot \;M_{R}\;\cdot \;M_{T}\times N_{T}\;\cdot \;M^{2}}.$$

The compressed sensing model can now be expressed in terms of the forward model A for the FMC data in (7) and the compression matrix Φ in (12). This model is written as

(13) $$y=\Phi \;\cdot \;\left(b+\tilde{n}\right)=\Phi Ax+n\in C^{N_{F}\;\cdot \;M_{R}\;\cdot \;M_{T}}.$$

Finally, given the prior knowledge that the ROI contains only a small number of flaws and therefore the reflectivity x is sparse, this imaging task can be carried out by solving the basis pursuit denoising problem [28]

(14) $$\min_{x}{∥y-\Phi Ax∥}_{2}^{2}+\lambda {∥x∥}_{1}.$$

Several methods exist for solving problems of the form (14). In this work, the Fast Iterative Shrinkage/Thresholding Algorithm (FISTA) [29] is employed.

### 4.2. FISTA

FISTA is a simple algorithm that allows problems like (14) to be solved iteratively. It addresses the data fidelity term ${∥\;\cdot \;∥}_{2}^{2}$ through gradient steps and the sparsity term ${∥\;\cdot \;∥}_{1}$ via soft thresholding. Further details are not provided here, as they can be found in the literature [29]. Some complications arise, however, as the forward model A quickly becomes too large to store in memory even for small ROIs and small values of *M*. In addition, the choice of the sparsity-promoting parameter λ is left to the user. These two challenges are addressed next.

Since the data fidelity term requires gradient steps, an estimate of the the model’s largest singular value is required in order to guarantee convergence. The largest gradient step size for which the algorithm remains stable is inversely proportional to the square of the largest singular value $\sigma_{1}$ of ΦA, meaning it is preferable to overestimate this quantity in exchange for slower convergence rather than underestimating it and having instability. A heuristic for the singular value $\sigma_{1}$ can be constructed based on the structure of the model matrix [13]. Assume that the horizontal resolution $\Delta_{x}$ is fine enough that every set of $N_{z}$ columns of ΦA is highly correlated to the next, and so the forward model’s rank is approximately $N_{z}$. For simplicity, assume also that the $N_{z}$ non-zero singular values are equal. Next, note that the Frobenius norm of a matrix is equal to the square root of the sum of the squared singular values of the matrix. Considering the previous assumptions, this yields ${∥\Phi A∥}_{F}=\sqrt{N_{z}}\sigma_{1}$. Due to the structure of the forward model, each column contains $M_{R}\;\cdot \;M_{T}$ echoes, and there are $N_{z}\;\cdot \;N_{x}$ columns in total. The Frobenius norm can then be substituted by a sum over the energies of the echoes. Note that the compressed model includes a product with an unnormalized DFT matrix that introduces a factor of $\frac{N_{T}}{2}$ to the pulse shape’s energy if no frequency subsampling takes place and the signal is analytic. If the transducer directivity is also ignored, these considerations yield an estimate given by

(15) $$\sigma_{1}\approx w_{1}\sqrt{\frac{N_{T}\;\cdot \;M_{R}\;\cdot \;M_{T}\;\cdot \;N_{x}}{2}}{∥h∥}_{2}.$$

In this expression, the vector $h\in C^{N_{T}}$ is a sampled version of the pulse shape $h^{\left(a\right)}\left(t\right)$ and the coefficient $w_{1}$ is a weight that can be manually tuned by trial and error if the heuristic for $\sigma_{1}$ is too small and FISTA fails to converge or is unstable.

The sparsity parameter λ can be chosen based on how the soft thresholding operation is applied in FISTA. Soft thresholding is performed on back-projections involving the compressed model ${\left(\Phi A\right)}^{H}$ and a residual vector. Because of this, if λ is set to ${∥\left(\Phi A\right)}^{H}{y∥}_{\infty }$, the solution will achieve maximum sparsity and be set to a zero vector. The parameter can then be chosen as a percentage of this quantity by introducing a coefficient, resulting in [5]

(16) $$\lambda =w_{2}{∥{\left(\Phi A\right)}^{H}y∥}_{\infty }.$$

The coefficient in (16) is chosen in the range $0\leq w_{2}\leq 1$, where $w_{2}=0$ results in a standard least squares problem and $w_{2}=1$ yields a zero vector as a solution.

## 5. Cramér-Rao Bound

Having incorporated the subsampling process into the model, the CRB can be employed to evaluate its impact. The CRB is a lower bound on the variance of any unbiased estimate of a parameter [21]. It also describes the behavior of estimators that are asymptotically unbiased, such as in the high SNR regime [30] and when parameter dictionaries are sampled finely [26]. As shown in [23] and observed in [22], the CRB of FMC data containing a single point-like scatterer inherently incorporates geometrical and signal-dependent information of the measurement scenario, favoring larger aperture areas and configurations in which more energy is collected. This makes it a suitable and widely applicable design metric for subsampling.

The CRB can be computed as the inverse of the Fisher Information Matrix (FIM) [21], which can be constructed from the model and its parameters. The models shown in (8) and (9) depend on the parameter vector $\xi ={\left[x_{d}\;\;z_{d}\;\;a_{d}\;\;\phi_{d}\right]}^{T}$ and the noise variance $\sigma_{n}^{2}$. By construction, both models have the same noise statistics, and so either choice of model obeys $y∼CN(\mu ,\sigma_{n}^{2}I_{N_{F}\;\cdot \;M_{R}\;\cdot \;M_{T}})$, with mean μ=Φb. The FIM can be computed from these quantities as

(17) J=2σn2Re∂μ∂ξTH∂μ∂ξT∈R4×4,

where Re{·} is the real part of a complex number. The expression shown in (17) is known as the Slepian–Bangs formulation of the FIM [21] and is applicable due to the model statistics. Alternatively, a slight variation of (17) is given by

(18) J=2σn2Re∂b∂ξTH(ΦHΦ)∂b∂ξT∈R4×4,

following directly from linearity and the independence of the compression matrix Φ from the parameter vector ξ.

Once the FIM is known, the CRB can be computed as $C=J^{-1}$. Not all of the quantities in C are relevant, as it contains the nuisance parameters $a_{d}$ and $\phi_{d}$. Based on the chosen order of the parameters in the definition of the parameter vector ξ, focus is restricted to the upper left quadrant of C given by

(19) $$\begin{array}{c} \tilde{C}=\left[\begin{array}{cc} C_{11} & C_{12}\\ C_{21} & C_{22} \end{array}\right], \end{array}$$

in which $C_{m,n}$ is the scalar element at the *m*th row and *n*th column of C. The main quantities of interest are then the CRB of the *x*-coordinate of the scatterer which is given by $\mathrm{CRB}\left\{x\right\}=C_{11}$, the CRB of the *z*-coordinate $\mathrm{CRB}\left\{z\right\}=C_{22}$, and their sum $\mathrm{Tr}\{\tilde{C}\}$, where Tr{·} is the trace of a matrix. The evaluation of these quantities can be done numerically and is not addressed further in this work.

## 6. Sparse Array Design

Now that the model and reconstruction procedures have been established, the choice of $\Phi \;=\;S_{s}⋄\;S_{f}$ remains. The approach explored in this work is the same as in [23], where the design procedure is defined as a minmax problem of the form

(20) $$\min_{S_{s},S_{f}}\;\;\max_{\left\{x_{d}\right\}}\;\;\mathrm{Tr}\left\{\tilde{C}\right\}$$

with maximization carried out over a discrete set of *x*-coordinates $\left\{x_{d}\right\}$ within the ROI. The motivation behind this formulation of the subsampling pattern design task is the interpretation of minmax problems. They yield the best performance in the worst case scenario, which makes them attractive approaches in spite of their complexity [31]. A few considerations can be taken in order to simplify the problem. Note that maximization occurs only with respect to the *x*-coordinates within the discretized ROI: it was previously shown in [23] that the CRB achieves its largest values immediately under the array (in the near field) and far away from it (in the far field where *z* is larger than the maximum spacing between active transducers). In settings for which the ROI is known to lie at a distance larger than the aperture of the array, it suffices to take $z=z_{\max}$ in the ROI to maximize the CRB with respect to *z*. Additionally, this framework allows practically inspired constraints to be enforced. The solution set can be, for example, restricted to include solutions where the active Tx element in a measurement cycle cannot be used for Rx, avoiding self-interference artifacts. Beyond this, the complexity may still be too large, making greedy algorithms a reasonable (although suboptimal) solution approach [17,20].

### 6.1. Constant Rx

Depending on the spatial subsampling approach, a different solution approach can be taken. In what follows, solution approaches are described and compared in terms of the number of operations required to find the compression matrix. Because the implementation of the CRB is flexible, the number of operations is given in terms of the number of maximizations. This shifts focus onto the search over the Tx and Rx selection matrices in order to build $S_{s}$ from (11), recalling that this expression is valid both for the constant Rx and the varying Rx approaches. In the following scenarios, it is assumed that the frequency subsampling matrix $S_{f}$ has already been generated, and so the spatial subsampling matrix is optimized taking each channel’s spectral samples into account.

Some auxiliary terms and operations are defined which are needed in order to fully specify the measurement setting. The first of these is the set of *x*-coordinates $x_{d}$, denoted as $\left\{x_{d}\right\}$ with cardinality $|\left\{x_{d}\right\}|=N_{x}$, where the defects can potentially be located. Similarly, the *x*-coordinates of the ULA sensors are collected in the set $|\{x_{k}\}|=M$. Additionally, the sets $S_{T}$ and $S_{R}$ are defined as the sets of selection matrices with the correct number of active elements in Tx $\left(M_{T}\right)$ or Rx $\left(M_{R}\right)$, respectively. The operator A∖B yields the set A excluding the elements in the set B, while A∪B is the union of the sets. For clarity, the upper left quadrant of the CRB $\tilde{C}$ is written as a function of the parameters it depends on as the optimization procedure is carried out. Finally, conditions such as the aforementioned requirement of avoiding using the same array element for Tx and Rx in the same measurement cycle are denoted as a boolean function $q(S_{s})$ whose value is one if the conditions are satisfied and zero otherwise.

When there is only one Rx selection matrix $S_{R}$, it is still reasonable to use brute force and do a an exhaustive search for moderately small arrays (e.g., *M* ≤ 16). From the number of selection matrices to be tested, the number of maximizations necessary for an exhaustive search is MMRMMT, where ab denotes *a* choose *b*. The exhaustive search consists simply of iterating over the sets $S_{T}$ and $S_{R}$ and keeping the best solution, i.e., the one that achieves the minmax. This design approach is illustrated in Algorithm 1 and corresponds to the method from prior work [23].
**Algorithm 1:** Design via Exhaustive Search 
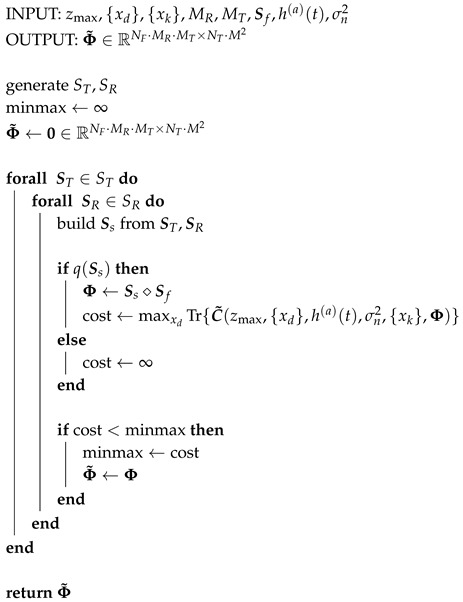


### 6.2. Varying Rx

An exhaustive search quickly becomes intractable in the varying Rx scenario, as the number of maximizations is now MMTMMRMT. In light of this, a greedy approach is adopted, and additional auxiliary terms are introduced. One Tx element $g_{T}\in \left\{1,\;2,\;\cdots ,\;M\right\}$ is tested at a time. For the Tx element under test, the best set $G_{Rc}$ of $M_{R}$ receivers is constructed by selecting, one at a time, the elements that reduce the minmax the most. Then, the Tx element and its Rx set $G_{Rc}$ for which the minmax is the smallest, which are denoted by a superscript *, are added to the final Tx set $G_{T}$ and Rx set $G_{R}$. Tx elements are then tested in the same way until all $M_{T}$ transmitters and $M_{R}\;\cdot \;M_{T}$ receivers have been chosen. The procedure is split into two parts: the Tx selection is depicted in Algorithm 2, and it makes use of Algorithm 3 in the form of function β(·) to choose $G_{Rc}$ at every step.

Approximately $M^{2}\;\cdot \;M_{R}\;\cdot \;M_{T}$ maximizations are needed; however, enough Tx and Rx elements must be pre-specified before the algorithm begins so that the FIM is invertible in the first iteration. This is because a minimum of two channels are needed in order to spatially resolve a defect in a 2D scenario. In this work, this greedy approach is initialized with a given Tx element $g_{T0}$ and an Rx element $g_{R0}$. This means the number of maximizations increases to approximately $M^{4}\;\cdot \;M_{R}\;\cdot \;M_{T}$ if all initial conditions are to be tested.
**Algorithm 2:** Greedy Tx Design 
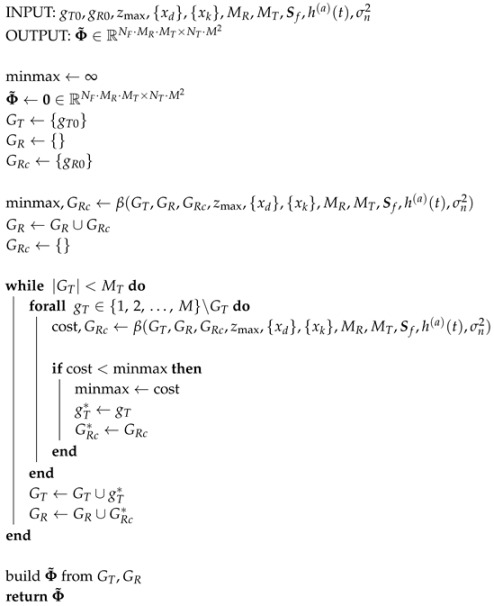
**Algorithm 3:** Greedy Rx Design (β(·)) 
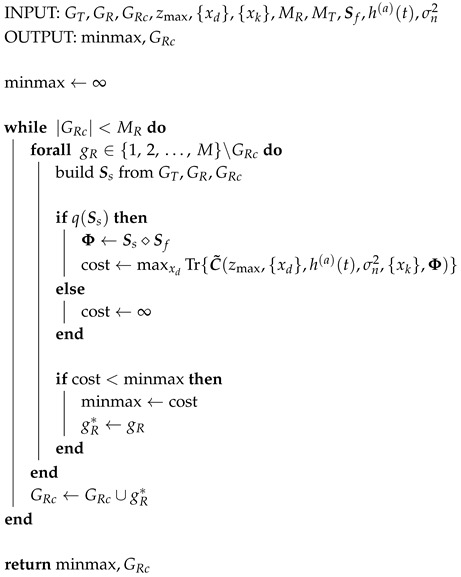


### 6.3. Fourier Subsampling

Reconstructions from frequency subsampled synthetic aperture data are discussed in [27], where strategies for the selection of the Fourier coefficients are also discussed. In this work, the so-called max and energy based frequency subsampling approaches are tested in tandem with the constant and varying Rx spatial subsampling approaches. Both the max and energy based frequency selection strategies depend on prior knowledge of the pulse shape in the medium. In practice, the pulse shape is not exactly known and may be different for each echo. However, a sample waveform can be obtained and used as a reference, e.g., by observing a back wall echo in a defect-free region of the specimen under test, or using a test specimen made of the same material as the specimen of interest. This reference waveform takes the place of the vector $h\in C^{N_{T}}$, i.e., the sampled version of $h^{\left(a\right)}\left(t\right)$. Recalling that the signal is analytic, a ”full” DFT matrix $F_{0}\in C\frac{N_{T}}{2}\times N_{T}$ allows the calculation of the pulse shape spectrum $F_{0}h\in C^{\frac{N_{T}}{2}}$, providing the necessary prior information.

The max strategy consists of selecting the $N_{F}<\frac{N_{T}}{2}$ elements of $F_{0}h$ with the largest absolute value, yielding the maximum possible energy. If the pulse spectrum has a single mode, this strategy results in a small bandwidth located near the center frequency of the pulse shape. This selection strategy is deterministic, and since a single pulse shape is assumed in the present model, every channel uses the same set of Fourier coefficients given by the matrix $F\in C^{N_{F}\times N_{T}}$ containing the $N_{F}$ rows of $F_{0}$ that yield the most energy when applied to the pulse shape.

The energy based strategy addresses the potentially small bandwidth of the max strategy, in exchange for reduced energy. In this approach, the frequency samples are chosen at random, but a higher probability of selection is assigned to samples with more energy. The normalization given by $F_{0}h/{∥F_{0}h∥}_{1}$ ensures the sum of the magnitudes of the pulse spectrum samples is one, after which it can be treated as a Probability Mass Function (PMF). Based on this PMF, a set of $N_{F}$ samples can be chosen by sampling without replacement. The selection process is random, and each channel has its own, possibly unique, set of frequency samples denoted by $F_{i}\in C^{N_{F}\times N_{T}}$.

## 7. Experiment

In order to compare the different spatial and frequency subsampling approaches, their compression matrices are paired with FISTA to yield reconstructions of a simulated FMC data set. The comparison is based on the performance of FISTA when reconstructing the underlying reflectivity x in a ROI at a fixed compression rate. This means that $N_{F}$, $M_{R}$, and $M_{T}$ are specified, and compression matrices using all four combinations of spatial and frequency subsampling approaches are constructed following (20). Additionally, sample reconstructions of measurement data are provided for qualitative evaluation.

### 7.1. Simulation Scenarios

**Figure 3:**
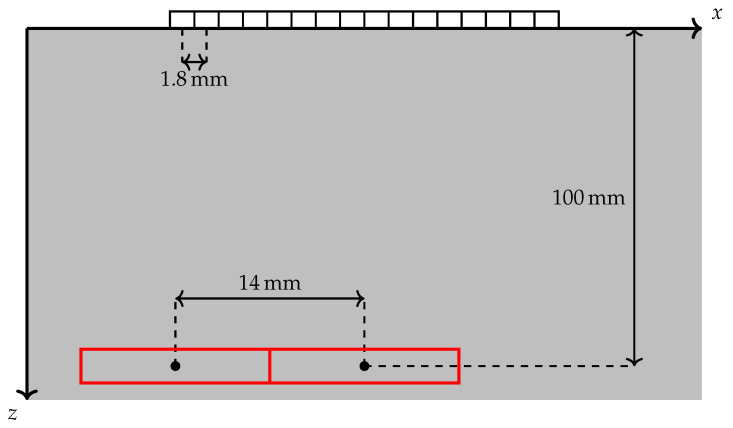
Illustration of the simulation setting. The *x*-axis is scaled up for clarity. The red rectangles are regions of interest of size 14 mm×10 mm where reconstructions are carried out.

Two simulation scenarios are illustrated in Figure 3. A homogeneous, isotropic specimen with a constant speed of sound $c=6300\;{m\;s}^{-1}$ is simulated. The sampling frequency is $f_{s}=40\;M\mathrm{Hz}$, and the measurements contain $N_{T}=1525$ time samples. A ULA with M=16 elements is represented by the rectangles above the *x*-axis. The spacing between the centers of consecutive sensors, or pitch, is $p_{x}=1.8\;mm$, and the opening angle is set to $\theta_{0}=32{}^{\circ }$. Two point-like scatterers are present in the medium, which is assumed to extend infinitely along ±x and +z. Their depth is z=100 mm. One of the scatterers is located under the ULA’s axis of symmetry at x=0 mm, while the other is placed at the leftmost edge of the array at x=−14 mm. The red boxes in the figure represent the ROIs in which reconstructions are carried out with FISTA. These regions are 14 mm wide and 10 mm tall with no overlap between them (although they are immediately adjacent to one another).

The resolution is $\Delta_{x}=360\;\mu m$ along the horizontal axis and $\Delta_{z}=78.75\;\mu m$ along the vertical axis. In each subsampling configuration, complex noise with a variance of $\sigma_{n}^{2}=0.0009$ is added in the time domain. Then, 20 reconstructions with different noise realizations but a fixed compression matrix are averaged. Reconstructions are obtained using 50 iterations of FISTA with $w_{1}=3$ and $w_{2}=0.45$. $M_{T}=3$ Tx elements and $M_{R}=4$ Rx elements are used, while the number of frequency coefficients is $N_{F}=31$ in one test scenario and $N_{F}=61$ in another. The computation of the compression matrix considers $\left\{x_{d}\right\}$ to contain all the points under the array instead of being limited to those within the ROIs, as the locations of defects cannot always be known a priori.

Some comments regarding the aforementioned parameters are offered: The number of iterations was chosen such that small increases yielded no noticeable improvement in the reconstruction quality when visually inspected. Values for $M_{T}$, $M_{R}$, and $N_{F}$ were chosen so that a large compression rate is obtained, but reconstructions are still reasonable (i.e., not dominated by artifacts). Regarding the hyper parameters $w_{1}$ and $w_{2}$, they are chosen through trial and error by making them as small as possible to reduce the number of iterations, yet not so small that FISTA fails to converge. These hyper parameters can also be computed optimally through data-driven approaches by, for example, unfolding the iterative reconstruction procedure into a neural network [32], assuming that the inability to store the model matrix can be circumvented. Reconstructions are, in general, not very susceptible to small changes in the parameters addressed in this note. Furthermore, they are often left up to the choice of the operator. In this work, they instead define the overall scenario that constrains the compression matrix design and image reconstruction procedure, and are therefore only tangent to the study at hand.

**Figure 4:**
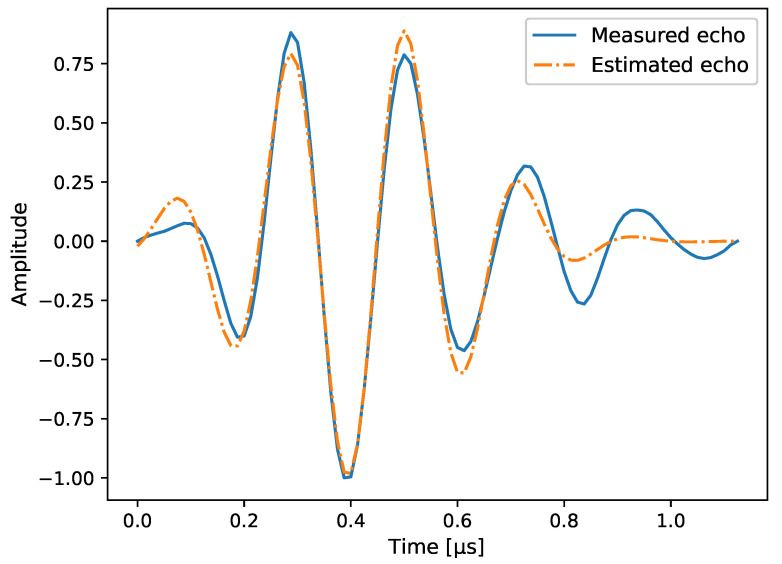
Illustration of a pulse shape taken from the back wall echo of a real aluminium specimen. Its envelope has been normalized.

The pulse shape $h^{\left(a\right)}\left(t\right)$ used in the simulations is shown in Figure 4. It has been taken from real measurement data, and corresponds to the back wall echo of an aluminium specimen with the aforementioned speed of sound. Since an analytical expression is not available, the time delays $\tau_{i,j}\left(x_{d},z_{d}\right)$ are applied to $F_{0}h$ and the pulse shape is then transformed back into time domain. In order for the CRB to be computed, though, a differentiable expression is needed. To this end, the pulse shape is approximated with a Gabor function of the form

(21) $$\begin{array}{c} h\left(t\right)=\exp\left(-\alpha t^{2}\right)\;\cdot \;\cos\left(2\pi f_{c}t+\phi_{0}\right) \end{array}$$

whose analytic representation is taken afterwards. The parameters for this pulse shape are the bandwidth factor $\alpha ={\left(3.8143/\mu s\right)}^{2}$, its center frequency $f_{c}=4.5471\;M\mathrm{Hz}$, and a phase shift of $\phi_{0}=-2.6143\mathrm{rad}$ which is independent of the previously defined $\phi_{d}$ of each point-like scatterer.

**Figure 5:**
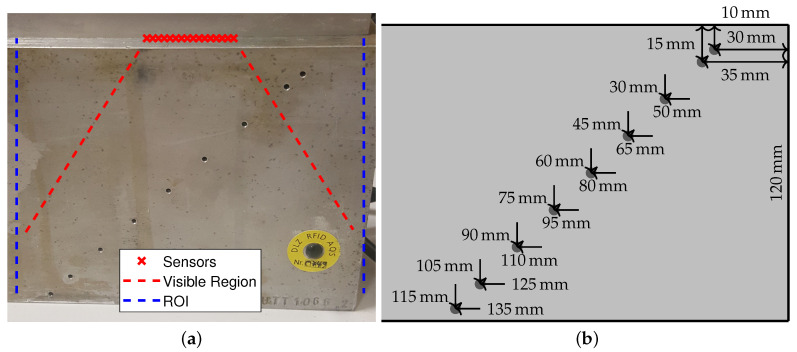
Aluminium specimen with 2 mm diameter side drilled holes. (**a**) Picture of the specimen and array with markers showing the approximate location of the active aperture, visible region, and reconstruction region. Adapted from [13]. (**b**) Locations of the holes relative to the upper right corner of the specimen.

A qualitative performance test is performed on a data set collected from the aluminium specimen in Figure 5, from which the pulse shape in Figure 4 was taken. The parameters match those from the simulation setting in Figure 3. It should be noted that although seven of the side drilled holes fall roughly within the region that is visible by the ULA, their visibility depends on the number of channels in which their time traces appear, as well as the DOA and DOD. Flaws beyond the horizontal aperture of the array are inherently more difficult to detect; indeed, the two leftmost and the rightmost holes are not visible when using standard TFM on the complete data set. Because of this, the maximization over *x* in (20) includes only values of $x_{d}$ directly under the array.

### 7.2. Comparison Metrics

The reconstructions are compared in terms of their Array Performance Indicator (API) and the aspect of the vertical and horizontal cross sections of the main lobe of their Point Spread Function (PSF). Although FISTA should yield a single non-zero pixel, the presence of noise and failure to converge in a specified number of iterations results in a characteristic PSF. The API conveys the normalized cross section area of a PSF at a given threshold value, and can be computed as [1]

(22) $$\begin{array}{c} \mathrm{API}=\frac{N_{\mathrm{thresh}}\;\cdot \;\Delta_{x}\;\cdot \;\Delta_{z}}{\lambda_{c}^{2}}, \end{array}$$

where the threshold value value can range from zero to one for an amplitude-normalized PSF, $N_{\mathrm{thresh}}$ is the number of reconstruction pixels at or above the threshold, and $\lambda_{c}$ is the wavelength. For the pulse shape in Figure 4, the center frequency is $f_{c}=4.5471\;M\mathrm{Hz}$ and the corresponding wavelength is $\lambda_{c}=1.385\;mm$.

## 8. Results

### 8.1. Simulated Scenarios

In the following plots, a shortened nomenclature is adopted for brevity. In the legends, Full denotes no spatial nor frequency subsampling. The letters V. and C. stand for varying Rx and constant Rx, respectively. The ending max refers to the deterministic frequency subsampling strategy in which samples with the largest amplitude are chosen, while PMF refers to the energy based approach that uses the spectrum of the pulse shape as a PMF from which coefficients are drawn.

**Figure 6:**
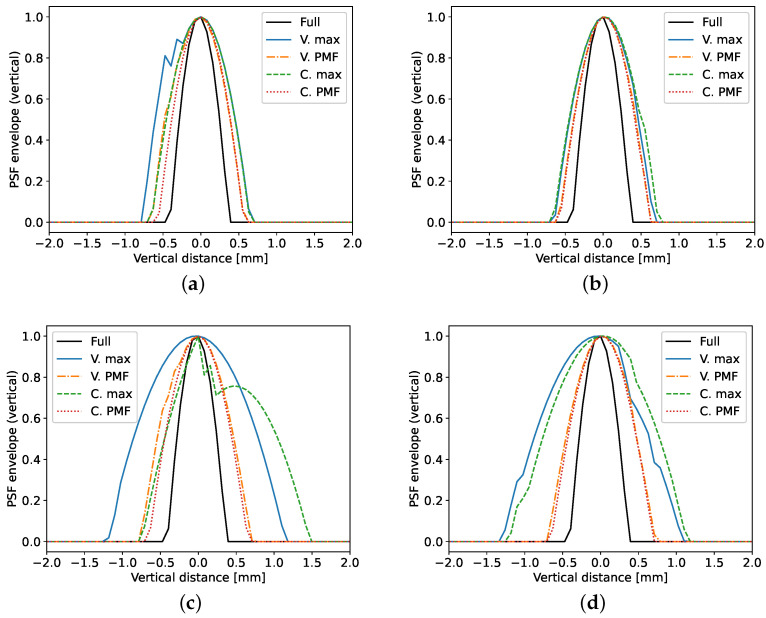
Vertical cross section through the maximum of the PSF. Each tile corresponds to one of the simulation scenarios: (**a**) defect on axis of symmetry with $N_{F}=61$, (**b**) left defect with $N_{F}=61$, (**c**) defect on axis of symmetry with $N_{F}=31$, (**d**) left defect with $N_{F}=31$.

The vertical slices of the PSFs are depicted in Figure 6. A comparison between the first and second row of figures reveals that the number of frequency coefficients affects the axial resolution of the reconstructions. Closer inspection reveals that this is only the case for the frequency selection strategy that chooses the largest Fourier coefficients, which results in a smaller bandwidth. Image (**c**) highlights the usefulness of the energy based strategy, as it the small bandwidth of the max strategy can make it difficult to determine the size and location of the defect as in the C. max plot. On this axis of the PSF, the choice of channel selection strategy has little impact. This is beneficial, since the greedy varying Rx approach can be used at a reduced computational cost.

**Figure 7:**
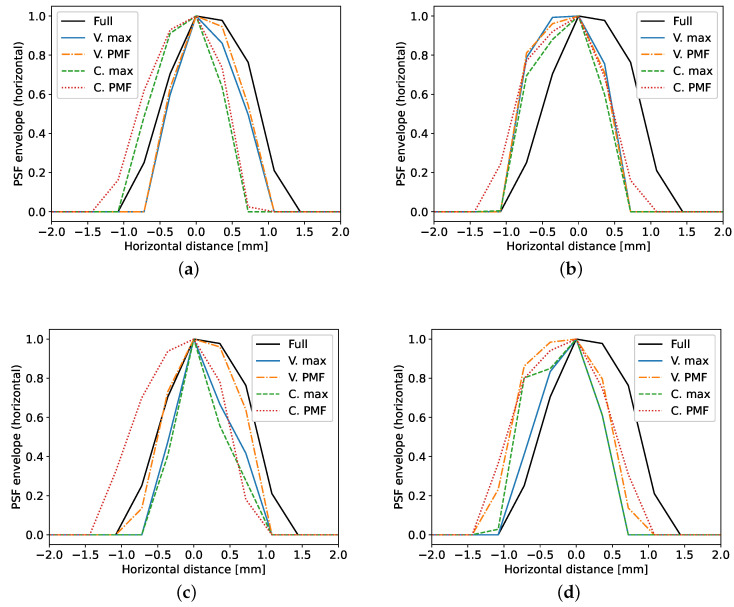
Horizontal cross section through the maximum of the PSF. The subfigures portray the scenarios under test: (**a**) defect on axis of symmetry with $N_{F}=61$, (**b**) left defect with $N_{F}=61$, (**c**) defect on axis of symmetry with $N_{F}=31$, (**d**) left defect with $N_{F}=31$.

Figure 7 contains the horizontal slices of the same PSFs. The images therein show that the specific choice of Tx and Rx channels impacts the PSF shape along this axis. In spite of the shape difference among the main lobes, the images appear to show that the horizontal resolution is improved when using compression matrices as opposed to taking all of the channels. In order to accurately convey how the size of the PSFs changes in each setting, the API is used next.

**Figure 8:**
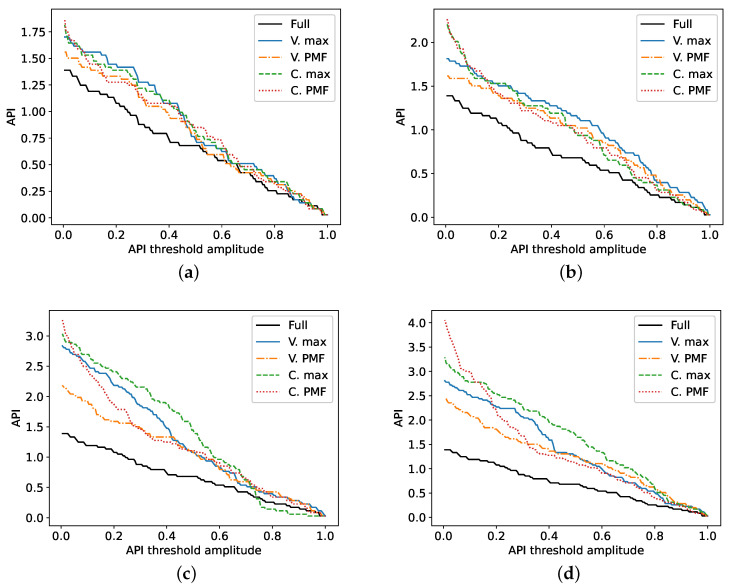
API of the PSFs at different threshold levels. As before, each plot corresponds to one scenario: (**a**) defect on axis of symmetry with $N_{F}=61$, (**b**) left defect with $N_{F}=61$, (**c**) defect on axis of symmetry with $N_{F}=31$, (**d**) left defect with $N_{F}=31$.

The API of each PSF is shown in Figure 8. In the scenarios with $N_{F}=61$, all of the approaches yield similar results. Under less severe subsampling conditions, the PSFs are expected to gradually come closer to that of the Full scenario, and the compression matrix design approach becomes less important. Even so, the PMF approaches exhibit a lower API than their counterparts below a threshold of 0.5, hinting at a smaller cross section areas and sharper decay beyond this point. This trend becomes more apparent in the row corresponding to $N_{F}=31$, where the additional bandwidth results in a drastic improvement of axial resolution, as depicted in Figure 6. Moreover, the varying Rx spatial subsampling strategy paired with PMF frequency selection has a more consistent behavior in all four scenarios than the constant Rx strategy with PMF, whose API worsens quickly at low threshold values.

### 8.2. Measurement Data

**Figure 9:**
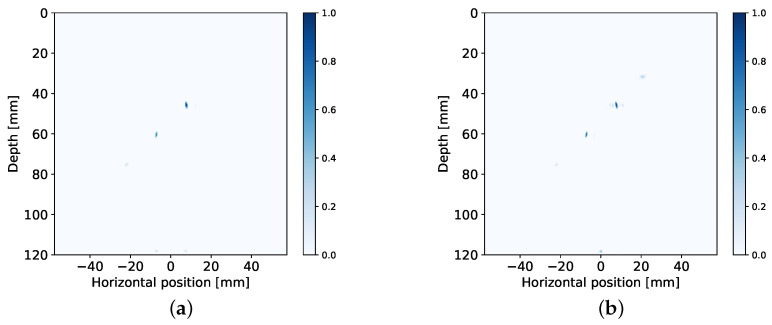

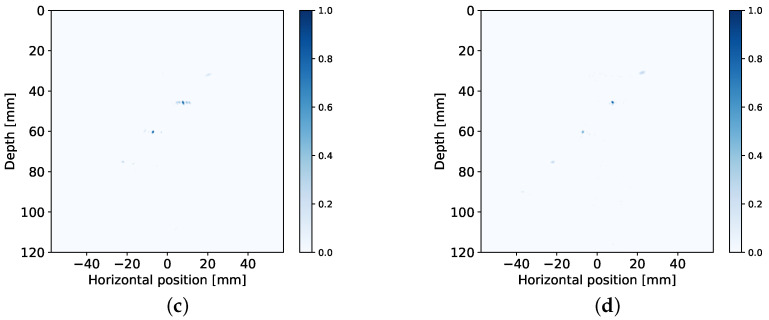
Reconstructions from measurement data of aluminium specimen with side drilled holes. The reconstructions illustrate the performance of different compression matrix design approaches under heavy subsampling: (**a**) constant Rx spatial subsampling paired with max strategy, $N_{F}=31$, (**b**) varying Rx spatial subsampling with max strategy using $N_{F}=31$, (**c**) varying Rx spatial subsampling with energy based approach and $N_{F}=31$, (**d**) reference reconstruction using more channels and frequency samples for comparison.

Reconstructions of the aluminium specimen are shown in Figure 9. For reference, a reconstruction computed at a lower compression rate (i.e., taking more channels and Fourier coefficients) is also provided. This reference is shown in Figure 9d, which was computed using $M_{T}=4$ Tx elements, $M_{R}=6$ Rx elements, and $N_{F}=81$ Fourier coefficients. The compression matrix for this reconstruction was designed using varying Rx spatial subsampling and the energy based approach for frequency subsampling. Additionally, the dynamic range of FISTA was increased by lowering $w_{2}$ to 0.1 and performing 100 iterations instead, resulting in an image where more of the side drilled holes are visible and artifacts are mitigated. This reference allows the visualization of the effect that each of the compression matrix design approaches has in extreme scenarios.

Comparing images (**a**) and (**b**) to (**d**), it can be observed that the shape of the side drilled holes has been stretched axially, matching the results obtained on simulated data. In the case of (**c**), the vertical resolution has improved. On the other hand, low amplitude artifacts are present in the vicinity of the side drilled holes, similarly to (**b**). The resulting PSFs are overall consistent between simulations and measured data, allowing the compression matrix design to be tested synthetically before deployment in a real world setting.

## 9. Discussion

Spatial and frequency subsampling can be used in order to reduce FMC measurement times and data volumes. By approaching imaging in post-processing from a compressed sensing standpoint, subsampling can be treated as a compression matrix acting on the complete FMC data and the underlying reflectivity information can be recovered. For spatial subsampling to reduce the measurement time, it must be performed in such a way that the number of measurement cycles is reduced [10,11,12]. Taking into consideration that this is to be done in such a way that it can be easily implemented, a special structure must be imposed on the compression matrix. In this work, compression matrices with a Kronecker and a Khatri–Rao structure have been studied. After noting the Kronecker structure to be a special case of the Khatri–Rao structure, the studied matrix structures can be categorized in four groups:

- A single set of receiving elements is shared by all transmitters and all channels use the same Fourier coefficients;
- Each transmitter has its own set of receivers and all channels use the same Fourier coefficients;
- A single set of receiving elements is shared by all transmitters but each channel uses a different set of Fourier coefficients;
- Each transmitter has its own set of receivers and each channel uses different Fourier coefficients.

The matrices were designed by solving a minmax problem using the Cramér-Rao bound of a single point-like scatterer as a cost function. In contrast with the exhaustive search approach from prior work [23], which is summarized in Algorithm 1, a much faster greedy approach is introduced in Algorithms 2 and 3. The matrices were tested on synthetic and measurement data in order to compare the approaches proposed in this paper versus the more rigid approach from [13]. The usage of different receivers by each transmitter does not yield improvements when compared to the case in which the transmitters share a common set of receivers. However, the former setting is based on a suboptimal solution found through a greedy algorithm which is faster to solve than the latter, which required an expensive exhaustive search. The varying receiver approach has the attractive advantage, then, of scaling better to larger scenarios with more sensors. Frequency subsampling approaches boil down to a trade-off between vertical and horizontal resolution. Following a strategy in which the samples with the largest magnitude are kept will often result in a small bandwidth that affects vertical resolution. In contrast, using the pulse shape spectrum as a probability mass function allows the coefficients to be chosen differently for each channel. Furthermore, the non-adjacent samples cover a larger bandwidth and yield an evident gain in vertical resolution under heavy compression settings. In exchange, artifacts are present in the horizontal axis and reconstruction is made difficult due to the reduction of energy. When these effects are quantified using the array performance indicator, a combination of varying receivers and randomly chosen Fourier coefficients performs the best, and the design of such compression matrices is manageable using greedy algorithms.

## 10. Conclusions

In this work, compressed sensing via spatial and temporal subsampling has been studied in the context of Full Matrix Capture data for Ultrasound Non-Destructive Testing. Since subsampling in transmission, reception, and frequency has been decoupled, designing the corresponding compression matrices is a daunting combinatorial task. To this end, a two stage greedy optimization algorithm has been presented. Comparisons on both measurement and synthetic data show that the proposed approach outperforms more rigid compression matrices in which subsampling is coupled, in spite of using a greedy suboptimal solution in the former and an optimal solution for the latter. The design approach is then tractable and easily applicable to real measurement settings, as the hardware requirements for subsampling are limited to programmable switches and frequency domain sampling.

## Abbreviations

The following abbreviations are used in this manuscript:

API	Array Performance Indicator
CRB	Cramér-Rao Bound
DFT	Discrete Fourier Transform
DOA	Direction of Arrival
DOD	Direction of Departure
FIM	Fisher Information Matrix
FISTA	Fast Iterative Shrinkage/Thresholding Algorithm
FMC	Full Matrix Capture
PMF	Probability Mass Function
PSF	Point Spread Function
PWI	Plane Wave Imaging
Rx	Receiver or Reception
ROI	Region of Interest
SNR	Signal to Noise Ration
TFM	Total Focusing Method
Tx	Transmitter or Transmission
UCT	Ultrasound Computed Tomography
ULA	Uniform Linear Array
